# Osteogenic protein 1 in synovial fluid from patients with rheumatoid arthritis or osteoarthritis: relationship with disease and levels of hyaluronan and antigenic keratan sulfate

**DOI:** 10.1186/ar1947

**Published:** 2006-04-28

**Authors:** Susan Chubinskaya, Benjamin S Frank, Margaret Michalska, Bhavna Kumar, Charis A Merrihew, Eugene J-MA Thonar, Mary Ellen Lenz, Lori Otten, David C Rueger, Joel A Block

**Affiliations:** 1 Department of Biochemistry, Rush University Medical Center, Chicago, Illinois, USA; 2 Section of Rheumatology, Rush University Medical Center, Chicago, Illinois, USA; 3 Stryker Biotech, Hopkinton, Massachusetts, USA

## Abstract

The measurement of body fluid levels of biochemical markers in joint tissues has begun to provide clinically useful information. Synovial fluid (SF) plays an important role in articular joint lubrication, nutrition, and metabolism of cartilage and other connective tissues within the joint. The purpose of our study was to identify and characterize osteogenic protein 1 (OP-1) in SF from patients with rheumatoid arthritis (RA) or with osteoarthritis (OA) and to correlate levels of OP-1 with those of hyaluronan (HA) and antigenic keratan sulfate (AgKS). SF was aspirated from the knees of patients with either RA or OA and from the knees of asymptomatic organ donors with no documented history of joint disease. The presence of detectable OP-1 in SF was demonstrated by western blots with specific anti-pro-OP-1 and anti-mature OP-1 antibodies. Measurement of levels of OP-1, HA and AgKS was performed using ELISAs. OP-1 was identified in human SF in two forms, pro-OP-1 and active (mature) OP-1 – mature OP-1 being detected only in SF from OA patients and RA patients. Levels of OP-1 and HA were higher in RA patients than in OA patients and asymptomatic donors, while the level of AgKS was highest in SF from asymptomatic donors. Statistically significant differences were found between SF levels of OP-1 in RA and OA patients and between SF levels of AgKS among the three groups tested. The SF content of OP-1 tended to correlate positively with HA levels, but negatively with AgKS concentrations. In conclusion, the results of this study suggest that measurement of OP-1 in joint fluid may have value in the clinical evaluation of joint disease processes.

## Introduction

The measurement of body fluid levels of biochemical markers of structural or metabolic changes in joint tissues has begun to provide clinically useful information. Synovial fluid (SF) plays an important role in articular joint lubrication, nutrition and metabolism of cartilage and other connective tissues within the joint. Cartilage-derived molecules present in SF may be markers predominantly of biosynthetic changes or of degradative changes. Such markers of cartilage metabolism have been divided into two classes, direct markers and indirect markers [1].

Direct markers originate from cartilage structures and provide a measure of the responses of chondrocytes or changes that occur in cartilage. Among these is antigenic keratan sulfate (AgKS), a molecule found almost exclusively in aggrecan molecules within cartilaginous tissues [2,3]. AgKS is released when aggrecan is cleaved by proteolytic enzymes, whereupon the AgKS-bearing fragments may be measured in various body fluids. The indirect markers of cartilage metabolism, on the other hand, are found in many tissues and are produced by a variety of cell types [1]. These indirect markers include, but are not limited to, proteolytic enzymes, proteinase inhibitors, proinflammatory cytokines and matrix molecules, such as hyaluronan (HA), C-reactive protein, and so forth.

While they may not provide a reliable measure of intra-articular events, a number of studies have reported an association between the levels of certain markers in SF and joint changes in arthritic diseases [2-7], and have helped to identify markers that may have prognostic and/or diagnostic value in rheumatoid arthritis (RA) and osteoarthritis (OA).

Osteogenic protein 1 (OP-1), a member of the bone morphogenetic protein (BMP) family, is expressed by human adult articular chondrocytes and plays a crucial role in the maintenance of cartilage matrix integrity and the promotion of repair processes [8,9]. OP-1 has a potent anabolic effect on articular cartilage and other connective tissues: it stimulates the synthesis of major cartilage matrix components [10-12], it promotes matrix assembly [13], and it serves as an antagonist to the deleterious effects of catabolic mediators [14-16] without inducing chondrocyte hypertrophy and proliferation [10,11]. OP-1 gene expression and protein expression have been detected in all of the connective tissues of the joint – cartilage, meniscus, synovium, ligament, and tendon [17] – and there appears to be a negative correlation between autocrine OP-1 production and degenerative articular processes [18,19].

The objectives of the current study were: to characterize the OP-1 present in human SF; to compare levels of endogenous OP-1 protein in SF obtained from organ donors, from OA patients, and from RA patients using a validated ELISA [19]; and to correlate these levels with those of other validated biochemical markers of joint tissue metabolism, specifically AgKS [20] and HA [21]. In the present article, we provide the first report of the presence of OP-1 in the SF of asymptomatic organ donors, of OA patients, and of RA patients, and report statistical differences between the SF concentrations of this growth factor in OA patients and RA patients.

## Patients and methods

### Subjects

Synovial fluid was aspirated within 24 hours of death from the knee joints of 14 asymptomatic human organ donors with no documented history of joint diseases. This study, performed with assistance from the Gift of Hope Organ & Tissue Donor Network (Elmhurst, IL, USA), received institutional approval (ORA #00091901 approved on 3rd October 2000). Synovial fluid was also obtained with appropriate consent from 29 OA patients and 25 RA patients of the Rush Section of Rheumatology who were undergoing diagnostic or therapeutic arthrocentesis as part of their evaluation and therapy. The patient cohort covered a broad spectrum of age and disease severity (both RA and OA). Disease categories represent primary diagnoses as determined by the attending physician and were based on clinical and radiologic criteria (Table 1). OA was defined according to the classification criteria disseminated by the American College of Rheumatology [22]. Samples were centrifuged to remove cells and debris, divided into aliquots and were immediately frozen at -80°C.

### OP-1 antibodies

Four different antibodies were used for this study: two polyclonal antisera, R2854 (Stryker Biotech, Hopkinton, MA, USA) and sc-9305 (Santa Cruz Biotechnology Inc., Santa Cruz, CA, USA); and two mAbs, 1B12 (Stryker Biotech) and MAB 354 (R&D Systems, Minneapolis, MN, USA). All antibodies have been previously described and characterized [8,18,19,23]. The polyclonal antibody R2854 was raised in rabbits against the monomeric pro-domain of the OP-1 molecule and recognizes the OP-1 pro-domain. All other antibodies recognize the mature domain of OP-1: two mAbs, 1B12 and MAB354, were raised against the monomeric mature domain of OP-1 and a third, polyclonal antiserum (sc-9305), was raised against a 15-amino-acid synthetic peptide within the N-terminus of the mature OP-1 domain.

### OP-1 western blot analysis

Western blot analyses were performed with anti-pro-OP-1 (R2854) and anti-mature OP-1 (MAB354) antibodies. To optimize the methodology, SF samples were tested at different dilutions (1:5, 1:10, 1:100, and 1:1000) before and after enzymatic digestion with hyaluronidase (50 units/ml), with chondroitinase ABC (0.1 units/ml), or with a combination of both enzymes at 37°C for 90 minutes. Digestion with chondroitinase ABC was performed in the presence of a protease inhibitor cocktail. Samples were boiled for 5 minutes in a heat block and then loaded onto SDS-PAGE gels (12%) under reduced (with 2-mercaptoethanol) or nonreduced conditions, following which western blots were performed. For each sample, 30 μg protein was loaded.

**Figure 1:**
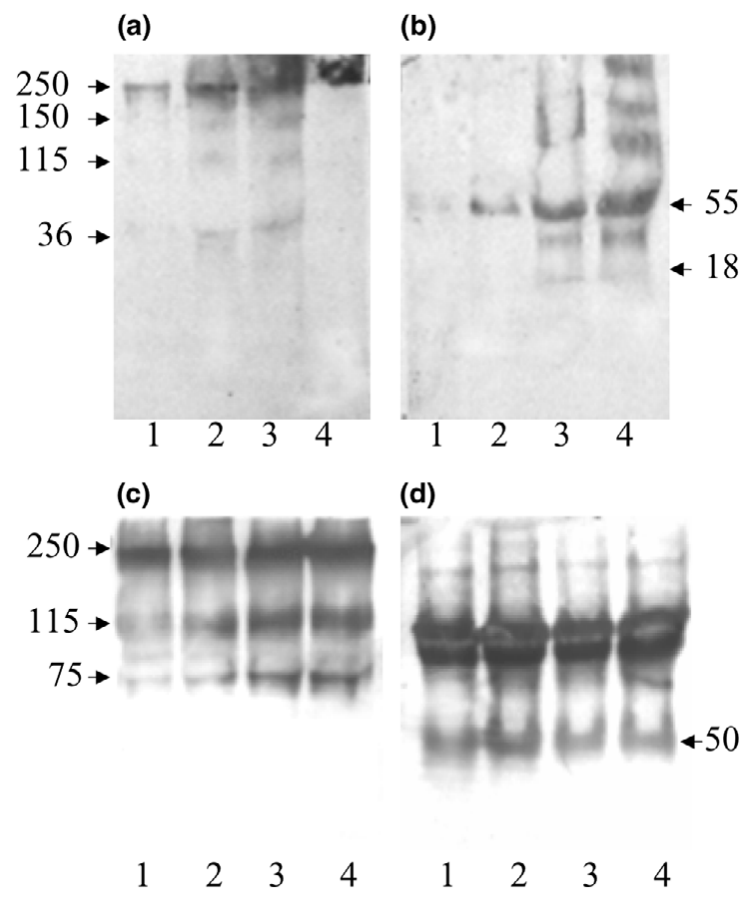
Western blots of synovial fluid samples. Representative western blots of a synovial fluid sample obtained **(a)**, **(b) **from a grade 0 normal asymptomatic organ donor or **(c)**, **(d) **from a patient with rheumatoid arthritis using an anti-mature osteogenic protein 1 (OP-1) antibody (MAB354) demonstrating the pro-form and mature form of OP-1 protein. (a) and (c) Nonreduced conditions, (b) and (d) reduced with β-mercaptoethanol. (a) and (b) Sample was loaded at different dilutions of sample buffer in the amount of 30 μl/lane: lane 1, 1:1000; lane 2, 1:100; lane 3, 1:10; and lane 4, 1:5. (c) and (d) Undiluted sample was digested with the following enzymes and then loaded onto each lane at 30 μg protein: lane 1, control, no treatment; lane 2, hyaluronidase (50 units/ml); lane 3, chondroitinase ABC (0.1 U/ml); lane 4, combination of hyaluronidase (50 units/ml) and chondroitinase ABC (0.1 U/ml). The numbers on gels represent the size of the protein bands of interest.

In the experiment described in Figure 1a,b, where serial dilutions of SF were tested, 30 μl each sample was loaded onto gels. To decrease nonspecific binding, blots were incubated with blocking solution containing 5% nonfat dry milk (Bio-Rad, Hercules, CA, USA) in Tris-buffered saline (TBS) with 0.05% Tween 20 (TBS/Tween) (Bio-Rad) for 1 hour at room temperature. The blots were then incubated with primary antibody at the manufacturers' suggested dilutions in 1:250 TBS/Tween for R2854 and MAB354 anti-OP-1 antibodies. Secondary antibodies were used, either ImmunoPure goat anti-mouse IgG (Pierce, Rockford, IL, USA) or donkey anti-rabbit IgG (Pierce) conjugated with horseradish peroxidase at 1:10,000 dilutions in TBS/Tween. The blots were developed with the SuperSignal West Pico Chemiluminescent substrate (Pierce) kit for western blotting. The specificity of binding was compared with the binding of the antibodies to recombinant pro-OP-1 or mature OP-1. Secondary antibodies were also tested for nonspecific binding. The densities of specific immunoreactive bands was scanned by the Fluor-S MultiImager (Bio-Rad) and quantified by the Quantity One Software program (Bio-Rad).

### Measurement of OP-1 by ELISA

For the quantitative assessment of OP-1 protein levels, aliquots of SF samples were first diluted 1:100 with TBS. OP-1 was subsequently detected with the OP-1 chemiluminescent sandwich ELISA developed in our laboratory, which recognizes all forms of the OP-1 protein that contain a mature domain [19]. For the sandwich ELISA, two anti-OP-1 antibodies, sc-9305 and 1B12, were used. Briefly, 96-well plates (Nalge Nunc, Rochester, NY, USA) were coated with the polyclonal anti-OP-1 antibody sc-9305 at 50 ng/well in TBS, pH 7.5, and were incubated overnight at 4°C. Nonspecific binding was blocked by adding 5% nonfat dry milk in TBS/Tween, pH 7.5 (200 μl/well) for 2 hours at room temperature.

To generate a standard curve, mature recombinant OP-1 (Stryker Biotech) was diluted in TBS/Tween at concentrations ranging from 10 ng/ml to 0.01 ng/ml. The diluted OP-1 standards and aliquots of SF (100 μl/well) were added to the plate and incubated for 1 hour at room temperature. The monoclonal anti-OP-1 antibody (1B12) in TBS, pH 7.5, was applied at a 1:1000 dilution (100 μl/well) and was incubated at room temperature for 1 hour. ImmunoPure goat anti-mouse IgG peroxidase conjugated antibody (100 μl/well; Pierce) in TBS, pH 7.5, was used at a 1:10,000 dilution as the detection antibody. The reaction was developed with Supersignal ELISA Femto Maximum Sensitivity Substrate (Pierce).

The data, expressed as relative light units, were obtained using the chemiluminescent ELISA plate reader Victor^2 ^(Wallac1420; Perkin Elmer, Turku, Finland). The OP-1 values obtained were normalized either to total volume or to the DNA content as determined by the pico green assay (Molecular Probes, Eugene, OR, USA).

### Measurement of AgKS by ELISA

AgKS in SF was quantified by a well-characterized ELISA [3,20] that includes an inhibition step and makes use of an anti-keratan sulfate mAb that is specific for a highly sulfated carbohydrate epitope present only at the nonreducing end of long keratan sulfate chains. The ELISA was performed at pH 5.3 to promote the steepness of the inhibition curves for both standard samples and SF samples. Reported values are equivalents of the International Standard of keratan sulfate purified from human costal cartilage [20]. The intra-assay variation was <3%, and the inter-assay variation was <4%.

### Measurement of HA by ELISA

Hyaluronan in SF was quantified by a previously well-described sandwich ELISA [21] that also made use of the aforementioned anti-keratan sulfate mAb to differentiate between the coated aggregating nonkeratan-sulfate-containing rat chondrosarcoma proteoglycans that capture HA and the keratan-sulfate-bearing aggregating proteoglycans that are subsequently added. The assay produces very similar values for HA levels in body fluids as five other immunoassays [24]. The reported intra-assay variation was <4%, and the inter-assay variation was <6%.

### Statistical analysis

All data were entered into a computer database and analyzed using Prism (version 3.0) from GraphPad Software (San Diego, CA, USA). All measurements were carried out in triplicate, with differences statistically evaluated (at the 95% confidence level) by one-way analysis of variance and nonparametric unpaired *t *test; *P *< 0.05 was accepted as significant. Quantitative data are presented through the text as the mean ± standard deviation. All graphs are displayed as the mean ± standard error of the mean.

## Results

### Demographics of three populations of samples

The SF used for this study was obtained from the knee joints of 14 asymptomatic organ donors with no documented history of joint disease (10 males and four females, 11 Caucasians and three African-Americans; mean age, 64.8 ± 13.2 years (range 40–92 years); mean Collins grade, 2.2 ± 1.31 (range 0–4) [25]). Patients with diagnosed OA (*n *= 29) and RA (*n *= 25) enrolled in this study were of both sexes (13 males and 16 females for the OA group, and seven males and 18 females for the RA group) and represented similar age categories – mean age for the OA group, 68.6 ± 15.1 years (range 37–87 years); mean age for the RA group, 51.4 ± 16.7 years (range 22–73 years) – and a similar ratio of racial origin.

### Detection of OP-1 by western blotting

**Figure 2:**
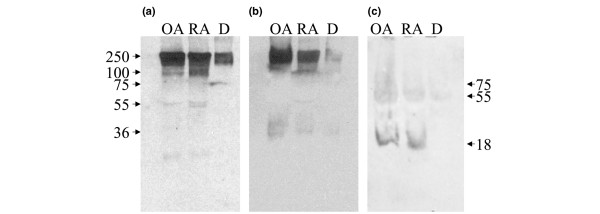
Western blots of synovial fluid samples from rheumatoid arthritis and osteoarthritis patients and organ donors. Representative western blots of synovial fluid samples from asymptomatic donors (D) and osteoarthritis (OA) and rheumatoid arthritis (RA) patients. **(a) **Nonreduced conditions with anti-pro-osteogenic protein 1 (OP-1) antibody (R2854). **(b) **Nonreduced conditions with anti-mature OP-1 antibody (MAB354). **(c) **Reduced gel with anti-mature OP-1 antibody (MAB354). Samples were diluted 1:100. The numbers on gels represent the size of the protein bands of interest. The same amount of protein was loaded onto each lane.

OP-1 was identified by western blots in all asymptomatic donor SF samples (Figures 1a,b and 2; MAB 354). It is worth noting that the MAB 354 and 1B12 anti-mature OP-1 antibodies used in this study recognize all forms of OP-1 that contain the mature OP-1 domain as part of its structure [8].

Under nonreduced conditions with anti-mature OP-1 antibody (MAB 354), the majority (approximately 70–80%) of OP-1 in the SF from asymptomatic donors was found as high-molecular-weight aggregates (about 250 kDa and above), although some OP-1 (approximately 15–20%) was present in the pro-form (molecular weight ~115 kDa) and very little (5–10%) was present in the mature active form (molecular weight ~36 kDa) (Figure 1a). Under reduced conditions (Figure 1b) the same antibody recognized a clear band migrating with a molecular weight of at least 55 kDa. This band represents the reduced form of the pro-OP-1 molecule. The light band at about 18 kDa, indicative of the reduced form of active OP-1, was barely detectable.

In order to identify the most appropriate conditions to analyze OP-1 in SF, serial dilutions of SF were performed (1:1000, 1:100, 1:10, and 1:5, Figure 1a,b). A 1/100 dilution of the SF samples yielded the clearest results. The small amount of the 18 kDa band (Figure 1b) suggests that OP-1 in asymptomatic donor SF is present predominantly as the pro-form. Undiluted (Figure 1c,d) OP-1 in RA samples remained mostly in the intact pro-OP-1 dimer form (molecular weight bands at 115 kDa and 250 kDa), even in the presence of reducing agents. It is not known whether OP-1 in SF is present as high-molecular-weight aggregates or is bound to other matrix macromolecules. Predigesting the samples with enzymes (hyaluronidase, chondroitinase ABC, or the combination of the two) did not alter the patterns of migration or the intensity of the OP-1 bands (Figure 1c,d).

In diluted samples from OA patients and RA patients (Figure 2), OP-1 was present not only in the pro-form (R2854), as was found in specimens from asymptomatic donors, but also in the active cleaved form (MAB 354). Immunoreactive bands at 75, 115 and 250 kDa probably represent uncleaved pro-OP-1, while bands at 36 kDa (unreduced conditions) and 18 kDa (reduced conditions) probably represent processed mature active OP-1 [8,25]. The distribution of OP-1 immunoreactive bands in SF was similar to that identified in human articular cartilage extracts [18,19].

### Quantification of OP-1 in SF by a sandwich ELISA

**Figure 3:**
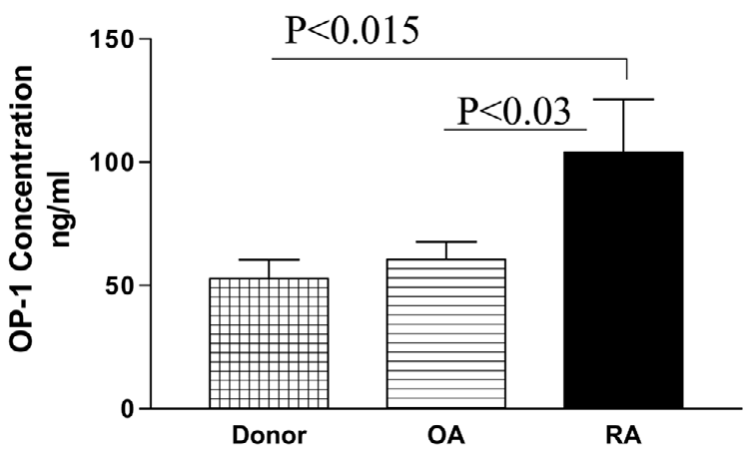
Content of osteogenic protein 1 protein in synovial fluid samples. The osteogenic protein 1 (OP-1) content of synovial fluid from asymptomatic donors (donor) and from osteoarthritis (OA) and rheumatoid arthritis (RA) patients detected by an OP-1 sandwich ELISA [19]. The data are presented as the mean ± standard error of the mean.

To detect OP-1 in SF by a well-characterized ELISA [19], aliquots of each SF sample were diluted 1:100. OP-1 was present at similar concentrations in SF from asymptomatic donors and from OA patients (donors, 52.8 ± 7.7 ng/ml; OA, 60.55 ± 7.17 ng/ml). Levels of this growth factor, however, were significantly higher in RA patients (116.9 ± 24.18 ng/ml; donors versus RA patients, *P *< 0.015; OA patients versus RA patients, *P *< 0.03; Figure 3). The asymptomatic donor group consisted of 14 SF samples that were aspirated from joints that, in certain cases, exhibited some degenerative changes (Collins grade varied from 0 to 4; Table 1) [26,27]. Although these donors were asymptomatic, some of them may represent patients with preclinical OA.

### Quantification of HA in SF by ELISA and correlation with OP-1 content

**Figure 4:**
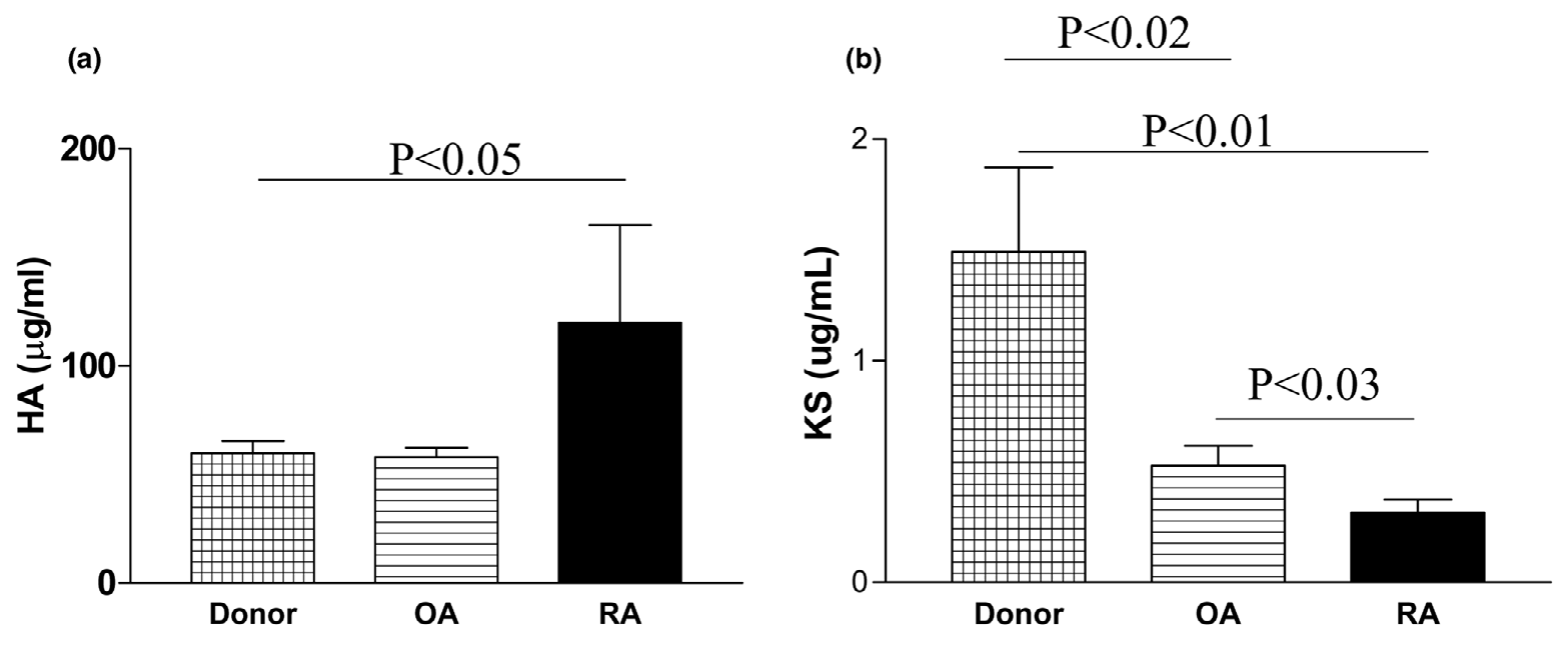
Concentration of hyaluronan and antigenic keratan sulfate in synovial fluid samples. The levels of **(a) **the hyaluronan (HA) concentration and **(b) **the antigenic keratan sulfate (KS) in synovial fluid from asymptomatic donors (donor) and from osteoarthritis (OA) and rheumatoid arthritis (RA) patients detected by an ELISA ([21] and [20], respectively). The data are presented as the mean ± standard error of the mean.

**Figure 5:**
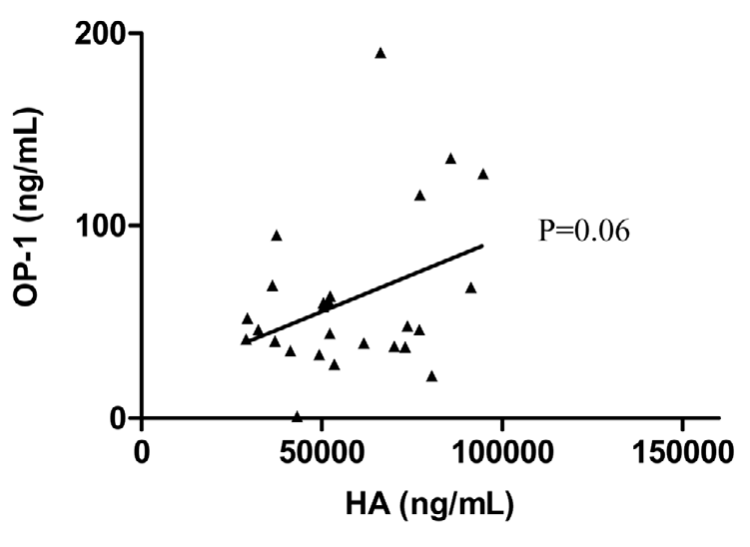
Correlation between osteogenic protein 1 and hyaluronan levels in synovial fluid samples from osteoarthritis patients. In the osteoarthritis group, there is a tendency for a positive correlation between synovial fluid osteogenic protein 1 (OP-1) and hyaluronan (HA) contents (*r*^2 ^= 0.1345, *P *= 0.0654).

Consistent with previous studies, synovial fluid levels of HA (Figure 4a) were also significantly higher in RA patients (119.8 ± 4.49 μg/ml) than in asymptomatic donors and patients with OA (59.75 ± 17.2 and 57.97 ± 2.15 μg/ml, respectively; donors versus RA patients, *P *< 0.05). Comparison of all the values for patient and asymptomatic donor groups suggested a possible trend for a positive correlation between SF levels of OP-1 and HA (*r*^2 ^= 0.05061, *P *= 0.12; data not shown). Further evaluation of this relationship within each group showed that the trend for this correlation was evident in asymptomatic donor samples (*r*^2 ^= 0.09717, *P *= 0.4142), but was especially evident in OA samples (*r*^2 ^= 0.1345, *P *= 0.0654) (Figure 5). There is one sample in the OA group that has the highest level of OP-1 and may perhaps be an outlier. The removal of this sample makes the positive correlation between OP-1 and HA in the OA group statistically significant (*P *< 0.045). No such trend was observed for SF samples obtained from RA patients (*r*^2 ^= 0.02600, *P *= 0.5818).

### Quantification of keratan sulfate in SF by ELISA and correlation with OP-1 content

The SF levels of AgKS were higher in asymptomatic donors (5.2 ± 2.67 μg/ml) than in OA patients (0.53 ± 0.46 μg/ml; donors versus OA patients, *P *< 0.02; Figure 4b), and they were lowest in RA samples (0.31 ± 0.23 μg/ml; donors versus RA patients, *P *< 0.01; Figures 4b). In marked contrast to what was observed for HA, levels of OP-1 tended to correlate negatively with levels of AgKS (data not shown), although no statistical significance was achieved.

## Discussion

The presence of measurable amounts of endogenous OP-1 in human SF was documented for the first time in the current study. At this time, the existence of other BMPs in SF has not been reported. Notably, the levels of endogenous OP-1 detected in SF from asymptomatic donor joints (about 50 ng/ml) were comparable with those extractable from normal articular cartilage (about 50 ng/g dry weight or about 150–200 ng/ml) [18,19]. Although we did not identify quantitative differences in the levels of endogenous OP-1 in asymptomatic donor SF relative to osteoarthritic SF, there were specific qualitative differences; whereas asymptomatic donor SF had no detectable or barely detectable active (mature) OP-1, the SF from OA joints had both pro-OP-1 and active (mature) OP-1. The absence of significant differences in the overall levels of OP-1 in asymptomatic donor and OA SF may be due to several factors. The 'asymptomatic donor' group consisted of a limited number of samples within which there were wide variations in age, sex, medical history, cause of death, and the morphological state of the joints. Moreover, although SF was obtained exclusively from asymptomatic organ donors without a prior history of joint disease, the majority of these joints displayed moderate degrees of morphological changes (Collins grade varied from 0 to 4), which may reflect the presence of pre-clinical OA conditions. Studies should thus be performed to distinguish between the concentrations of OP-1 in SF from truly normal joints (Collins grade 0–1) and from degenerative joints (Collins grade 2–4 and Mankin grade 5 and above) [28].

In contrast to the situation in normal cartilage matrix, the majority of the OP-1 resident in SF appeared to be in the pro-form. This raises an important question about the source of pro-OP-1 in SF and the proteins that OP-1 may be bound to. The latter issue is of particular interest to biotech companies that focus on the therapeutic applications of BMPs and their optimal formulations for delivery into the joints. Because OP-1 has been identified in all connective tissues of the synovial joint, the OP-1 found in SF could be derived from any or all of these tissues; this remains to be clarified. Another interesting finding is that OP-1 was present in the cleaved active form in the SF of OA patients and RA patients, suggesting that, at least in part, the active form of OP-1 could be generated during synovitis [29,30].

It is also possible that the cleavage and activation of OP-1 in joint disease reflects the action of proteinases induced by catabolic mediators active in RA and the late stages of OA. Our previous animal and human studies support this statement [23,31]. In a well-recognized animal model of OA, the intra-articular injection of chymopapain into the rabbit knee joint induced the activation of OP-1 in cartilage, which was detected by immunohistochemistry [31]. In addition, the activation and release of mature OP-1 protein in organ cultures of normal human adult articular chondrocytes treated with IL-1β was noted [23]. The finding that SF levels of OP-1 were higher in RA patients than in OA patients or asymptomatic donors is also consistent with recent reports that IL-1, which is present at higher concentrations in RA joints than in OA joints, is an effective modulator and/or stimulator of BMP-2 and OP-1 mRNA expression by normal and OA human articular chondrocytes [23,32,33]. These data are also in line with previous findings that documented an elevation of transforming growth factor beta in SF of RA patients [29]. Furthermore, we believe that elevated levels of OP-1 protein in RA SF may be due to the release of OP-1 residing in the extracellular matrix rather than to an increase in its synthesis. This belief is because matrix metalloproteinases activated by cytokines present in SF of RA patients induce the depletion of the extracellular matrix [34], thus promoting the release of growth factors bound to its latent domains or to the matrix components [35].

Interestingly, SF levels of OP-1 tended to correlate positively with levels of HA, but correlate negatively with levels of AgKS. In our studies, the highest concentration of HA was found in RA SF compared with asymptomatic donor and OA SF samples. This may primarily represent hypermetabolism in the synovium and other connective tissues, as reported by Thonar and colleagues [36], and could be the result of increased inflammation in RA joints. Higher serum levels of HA have been demonstrated to prognosticate the rapid destruction of cartilage and joints [37-39]. OP-1, BMP-2 and other growth factors have been shown by us and others [23,32,40] to increase in response to inflammation. The trend towards a positive correlation between SF levels of HA and OP-1 found in this study is therefore not surprising and may have a prognostic and/or diagnostic value for the inflammatory processes in articular joints.

The tendency for a negative correlation between OP-1 SF levels (anabolic factor) and AgKS content (a measure of the metabolic activity of the cells) supports the role of OP-1 in promoting anabolic responses and reducing catabolic events [14-16]. The elevated levels of AgKS could also suggest that cartilage matrix remodeling is higher in asymptomatic samples than in symptomatic samples. The majority of studies on SF markers have previously attempted to correlate levels of catabolic factors that promote or cause cartilage degradation with levels of fragments of matrix molecules that were purported to reflect primarily an enhancement in the turnover or degradation of cartilage or other connective tissues [4,5]. In the current study we attempted to compare parameters with opposite modes of action: comparing OP-1, which promotes anabolic, reparative processes, with factors that have been considered markers of degradative events (AgKS) or of predisposition to cartilage degeneration (HA). While no definitive statistical correlations were found, clear trends were observed for positive correlation between OP-1 and HA levels, especially for OA SF samples.

In the future, strict selection criteria for each experimental group and a larger sample pool may help to better understand whether the measurement of the SF level of an anabolic factor such as OP-1 may have prognostic or diagnostic value in arthritic conditions, especially when the analysis is performed in conjunction with measurement of well-accepted markers of cartilage anabolic or catabolic processes. The inclusion of other catabolic agents, such as proinflammatory cytokines and proteinases, in such analyses may provide a better understanding of the processes occurring in the articular joint.

## Conclusion

Taken together, the results of this study suggest that measurement of OP-1 in joint fluid may prove to be of potential value in the clinical evaluation of joint disease processes. The results also suggest that identification of the binding partners of OP-1 in the formation of high-molecular-weight aggregates may provide important information for the formulation and delivery of growth factors into target areas.

## Abbreviations

AgKS = antigenic keratan sulfate; BMP = bone morphogenetic protein; ELISA = enzyme-linked immunosorbent assay; HA = hyaluronan; IL = interleukin; mAb = monoclonal antibody; OA = osteoarthritis; OP-1 = osteogenic protein 1; RA = rheumatoid arthritis; SF = synovial fluid; TBS = Tris-buffered saline.

## Competing interests

The authors declare that they have no competing interests.

## Authors' contributions

SC, the principal investigator of the study, made substantive intellectual contributions to the conception, design, analysis, interpretation, and writing of the data. BSF was involved in the acquisition, analysis and interpretation of the data and in drafting the manuscript. MM was involved in sample acquisition and analysis of the data. BK was involved in the development and adaptation of the ELISA method for SF and in the acquisition of data. CAM was involved in data acquisition. EJMAT was involved in interpretation of the data and revising the manuscript critically for important intellectual content. MEL and LO were involved in data acquisition and editing the manuscript. DCR was involved in the interpretation of data and editing the manuscript. JAB made substantive intellectual contributions to the study.

## Figures and Tables

**Table 1:** Demographical representation of human subjects enrolled in the study

Group	Sample number	Age (years)	Sex	Race	Collins grade
Donors	1	66	Female	Caucasian	4
	2	69	Male	Caucasian	2
	3	54	Male	Caucasian	2
	4	74	Male	Caucasian	3
	5	79	Male	African-American	2
	6	67	Male	Caucasian	4
	7	67	Male	Caucasian	4
	8	52	Male	Caucasian	2
	9	67	Female	Caucasian	3
	10	40	Female	African-American	1
	11	54	Male	Caucasian	0
	12	92	Female	Caucasian	2
	13	73	Male	Caucasian	2
	14	53	Male	African-American	0
Osteoarthritis patients	1	84	Male		
	2	85	Male		
	3	81	Male		
	4	81	Male		
	5	82	Male		
	6	84	Male		
	7	53	Female		
	8	58	Male		
	9	60	Female		
	10	54	Female		
	11	60	Female		
	12	75	Female		
	13	81	Female		
	14	41	Male		
	15	75	Female		
	16	67	Female		
	17	64	Female		
	18	87	Female		
	19	71	Male		
	20	70	Female		
	21	53	Female		
	22	71	Male		
	23	82	Male		
	24	79	Female		
	25	37	Female		
	26	51	Male		
	27	81	Male		
	28	83	Female		
	29	40	Female		
Rheumatoid arthritis patients	1	47	Female		
	2	42	Female		
	3	73	Female		
	4	56	Female		
	5	26	Female		
	6	41	Male		
	7	62	Male		
	8	53	Female		
	9	66	Female		
	10	66	Female		
	11	26	Female		
	12	26	Female		
	13	73	Female		
	14	42	Female		
	15	34	Female		
	16	49	Female		
	17	70	Male		
	18	53	Male		
	19	52	Male		
	20	40	Female		
	21	68	Female		
	22	49	Male		
	23	76	Male		
	24	22	Female		
	25	72	Female		

Collins grade was assigned only to the cadaveric joints. Racial background of the osteoarthritis patients and of the rheumatoid arthritis patients was not available to us.
